# Non-redundant summary scores applied to the North American Rheumatoid Arthritis Consortium dataset

**DOI:** 10.1186/1753-6561-3-s7-s39

**Published:** 2009-12-15

**Authors:** Nathan D Pankratz

**Affiliations:** 1 Department of Medical and Molecular Genetics, Indiana University, School of Medicine, Indianapolis, Indiana 46202, USA

## Abstract

After performing a genome-wide association study, it is often difficult to know which regions to follow up, especially when no one marker reaches genome-wide significance. Researchers frequently focus on their top *N *findings, knowing that true associations may be buried deeper in the list. Others focus on genes or regions that have multiple markers showing evidence of association. However, these markers are often in high linkage disequilibrium with one another (*r*^2 ^> 0.80), which indicates that these additional markers are providing redundant information. I propose a novel method that identifies regions with multiple lines of evidence, by down-weighting the contribution of additional markers in proportion to pairwise linkage disequilibrium. I have used this non-redundant summary score in my analysis of the North American Rheumatoid Arthritis Consortium dataset released as part of Genetic Analysis Workshop 16. Three regions were identified that had a genome-wide empirical *p*-value less than 0.01, including one novel region on chromosome 20 near the KCNB1 and PTGIS genes.

## Background

Studies designed to identify genes contributing to complex diseases have been ongoing for many years, utilizing different study designs and methods with varied success. Recently, genome-wide association studies have become very popular, employing from hundreds of thousands to millions of single-nucleotide polymorphisms (SNPs). When analyzing such datasets, maintaining an acceptable level of type I error has meant either employing a Bonferroni correction or an accepted genome-wide significance threshold that requires a *p*-value of around 1 × 10^-8^. In many cases this has proven unattainable, even when genotyping several thousand samples. Researchers frequently have no other choice but to list their top findings and hope that an independent sample (which would not have to meet such a stringent threshold) can replicate them. Unfortunately, many of the top hits are indeed false positives and the true positives have slightly larger *p*-values. Finding those true positives might be helped by focusing on genes or regions that have multiple hits in them, similar to what is implemented in the computer program PLINK's linkage disequilibrium (LD)-based clumping procedure [1]. Unfortunately, due to the nature of genomic structure, this only provides support that the genotyping is accurate and does not reflect independent lines of evidence that the statistical association is anything more than random chance. Two SNPs with similar *p*-values that are in high LD (*r*^2 ^= 0.99 or even *r*^2 ^> 0.80) are simply providing redundant information.

This gets at one of the fundamental limitations of traditional association analyses, namely that it assumes that there is a single causative variant that was preserved on a single ancestral haplotype background. In practice, several variants may lead to the same relevant outcome, which in this case is usually the up- or down-regulation of the expression of some gene. The same sequence change may also have arisen independently on multiple haplotype backgrounds. Moreover, expression could be affected by independent variants that are scattered within the gene, its promoter and its 3' region, encompassing an area that can be anywhere from several thousand to over a million base-pairs. This is an argument for looking at genes or regions as a whole, while still keeping in mind that regions close together often exhibit LD. Here I use a method that generates a score for a region by summarizing all association results within its limits while weighting each marker's contribution based on its pairwise LD to other significant markers.

## Methods

### Subjects

I have analyzed the Problem 1 dataset that was provided for Genetic Analysis Workshop 16. This genome-wide association scan from the North American Rheumatoid Arthritis Consortium (NARAC) contains data from the Illumina 550 k chip for 868 cases and 1194 controls. The discrete trait (presence or absence of rheumatoid arthritis) is provided along with sex, DRB1 genotype status, number of shared epitope alleles, and two quantitative traits (for the cases only). NARAC has identified several DRB1 alleles that increase the risk for disease (0101, 0102, 0104, 0105, 0401, 0404, 0405, 0408, 0409, 1001, 1402, and 1406) and has indicated that a few alleles (0401, 0404, 0405, 0408, and 0409) increase risk more dramatically that the others.

### Quality assessment

Analyses were limited to those markers with a call rate of 95% or higher and a minor allele frequency (MAF) of 0.01 or higher. Fisher's exact test was employed to assess differential rates of missing genotypes based on disease classification (case or control), and SNPs with significantly different rates of missing genotypes (*p *< 0.0001) were removed from further analysis. SNPs demonstrating significant deviation from Hardy-Weinberg equilibrium (*p *< 0.000001) in the control sample were removed from further analysis. Many markers were flagged for multiple reasons and a total of 48,739 markers (8.9%) were removed in this way.

### Genome-wide association analyses

Logistic regression was used to generate *p*-values for each marker. Sex was not significantly associated with disease (*p *= 0.21) and therefore was not included as a covariate in the model. Number of shared epitope alleles (*p *= 1.6 × 10^-81^) and number of deleterious DRB1 alleles (*p *= 7.5 × 10^-102^) both showed strong evidence of association with disease and were included as covariates. PLINK was used to perform these analyses [1]. Pairwise LD was computed for all markers on a chromosome using the computer program Haploview [2].

### Non-redundant summary scores

Non-redundant summary (NRS) scores were computed for SNPs that met the threshold to be an index SNP (*p *< 0.001). NRS scores were generated as follows: all adjacent SNPs that were within a certain distance from the index SNP (150 kb each side) and that met inclusion criteria (*p *< 0.01) were flagged. These SNPs were sorted based on their *p*-value, and the -log_10 _of those *p*-values was summed sequentially (the one with the lowest *p*-value first). As each SNP was added to the score, it was multiplied by 1 minus the maximum pairwise *r*^2 ^value of any SNP already in the score (i.e., anything more significant). In this way, redundancy is minimized. The sum is then divided by the square root of the total number of SNPs in the region.

Empirical *p*-values were derived by permuting the phenotype (covariates and phenotype were linked to maintain their relationship) and repeating the process for each replicate. Ten thousand replicates were generated in this way. Genome-wide significance was determined by taking the number of replicates generating a maximum NRS score anywhere in the genome that met or exceeded the observed score and dividing that number by the total number of replicates. This algorithm was implemented using an in-house java application (source code available upon request).

## Results

**Figure 1:**
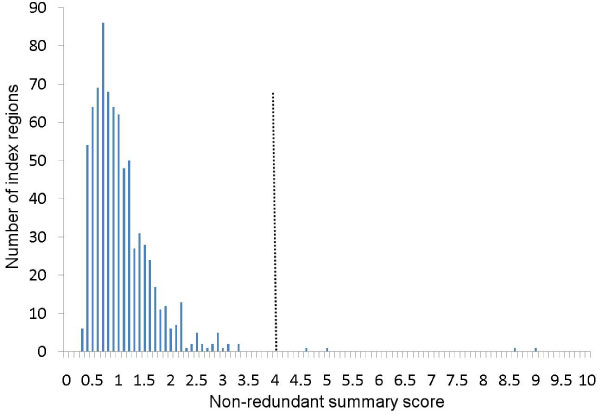
**Histogram of all non-redundant summary (NRS) scores**. The vertical dotted line indicates the empirically derived genome-wide significance threshold.

Association results are summarized in Table 1 and Figure 1. The strongest evidence of association was obtained to the regions on chromosome 6 known to be strongly associated with RA. Association was also noted on chromosomes 9 and 20. All of these regions were statistically significant (empirical genome-wide *p *< 0.05).

**Table 1:** Statistically significant (genome-wide) regions using 300-kb windows

Chr	Region	Index SNP	Position of index SNP	No. markers in region	NRS score	Empirical *p*-value
6	31289808-31589808	rs2523554	31439808	171	8.91	< 0.005
6	32295664-32595664	rs3129941	32445664	119	8.58	< 0.005
9	120670038-120970038	rs7037673	120820038	40	4.96	< 0.01
20	47360021-47660021	rs572845	47510021	67	4.52	< 0.01

## Discussion

Regions with multiple lines of evidence for association - i.e., association with different LD blocks and with markers of varying minor allele frequency - can be effectively summarized using the proposed NRS score. When applied to the NARAC dataset, three regions were significantly associated with affection status after adjusting for two covariates and correcting for multiple tests. The NRS scores for these regions were clearly distinct and separate from the rest of the distribution. This clear separation was not nearly as evident when SNPs were listed individually (pointwise) in a table. In this application, the SNPs that were individually most significant were located within the three regions identified by NRS, and no SNP that met genome-wide significance individually (*p *< 10^-8^) was outside of these three regions. This, however, is not always the case. This method was recently applied to a study of Parkinson disease (unpublished data) where no SNP individually met genome-wide significance. The NRS score, however, was able to single out a region that was statistically significant genome-wide.

The regions on chromosome 6, which contains the human leukocyte antigen (HLA) region and the DRB alleles, have already been well studied by NARAC. The other two regions also contain possible candidate genes. The area on chromosome 9 contains a single gene: *DBC1*, which is known for being deleted in breast cancers and is thought to play a role in cell cycle regulation. The novel region on chromosome 20 contains two genes: *KCNB1 *(a voltage-gated potassium channel gene) and *PTGIS*. *PTGIS *(Prostacyclin) is a particularly interesting candidate because it is a potent vasodilator and inhibitor of platelet aggregation. In contrast to these significant findings, the regions with NRS scores just below the significance threshold (3.0-4.0) all contained regions with multiple (five or more) small genes, indicating that such regions may somehow be inflating the score.

The parameters chosen prior to analyses (window size 300 kb, index threshold of *p *< 0.001, and inclusion threshold of *p *< 0.01) seem reasonable but are somewhat arbitrary. Using a smaller window size would increase the likelihood that a region with moderate *p*-values clustering in a small area would be more likely to have a higher NRS score. Requiring an index SNP to have a smaller *p*-value, on the other hand, decreases the number of regions that will be interrogated. This would increase the possibility that only regions that started off with a high pointwise rank would have a high NRS score, while clusters of slightly larger *p*-values would be ignored. Making the index parameter more liberal, on the other hand, would lead to empirical *p*-values growing to the point of insignificance. Decisions over parameters should ultimately be driven by what the researcher will ultimately trust in the end. For instance, if a researcher would not trust a region where the minimum *p*-value is only 0.01, then the index SNP threshold should be more stringent.

To test these theories, the algorithm was rerun using window sizes ranging from 50 kb to 500 kb, as well as using a more stringent index SNP threshold (*p *< 0.0001). The regions on chromosomes 6 and 20 were the only regions with significant SNPs using the more stringent index threshold. All four original regions contained significant SNPs when the window size varied. Only one new region with a genome-wide *p *< 0.05 was identified using the smallest window (25 kb on either side of rs4726160); though the *p*-value (*p *= 0.03) would not be considered significant after correcting for multiple models. This new region (chromosome 7: 151572154-151622154) spans a single gene, *MLL3*, which, like *DBC1 *on chromosome 9, is associated with a cancer phenotype.

Even though window size did not have a large impact on the conclusions reached, future directions will include modifying the score such that a specific window size need not be defined explicitly. For example, distance from the index SNP could be somehow incorporated into the model. Alternatively, this method could be adapted to be a gene-based approach, avoiding the issue altogether. Another future direction is to optimize the penalty function. Currently, the square root of the number of markers in the region is employed, since simply using the number of markers would be too conservative (because of the redundancy in the data that we are trying to filter out) and having no penalty at all would inflate type I error in regions where SNP arrays have a disproportionate number of SNPs, such as the HLA region. A more precise correction, perhaps based on recombination rate or LD distance, will be explored.

## Conclusion

The NRS score can be an objective guide to help researchers prioritize regions for follow up studies. Instances in which there is clear separation (as is the case with Figure 1) can also provide confidence that the researchers are not wasting valuable time and resources chasing red herrings.

## List of abbreviations used

HLA: Human leukocyte antigen; LD: Linkage disequilibrium; MAF: Minor allele frequency; NARAC: North American Rheumatoid Arthritis Consortium; NRS: Non-redundant summary; SNP: Single-nucleotide polymorphism.

## Competing interests

The author declares that they have no competing interests.

## Authors' contributions

NDP performed the statistical analysis and drafted the manuscript. The author has read and approved the final manuscript.
